# Proteomic and genomic integration identifies kinase and differentiation determinants of kinase inhibitor sensitivity in leukemia cells

**DOI:** 10.1038/s41375-018-0032-1

**Published:** 2018-04-07

**Authors:** Pedro Casado, Edmund H. Wilkes, Farideh Miraki-Moud, Marym Mohammad Hadi, Ana Rio-Machin, Vinothini Rajeeve, Rebecca Pike, Sameena Iqbal, Santiago Marfa, Nicholas Lea, Steven Best, John Gribben, Jude Fitzgibbon, Pedro R. Cutillas

**Affiliations:** 1 Cell Signalling & Proteomics Group, Centre for Haemato-Oncology, Barts Cancer Institute, Queen Mary University of London, London, UK; 2 Cancer Immunology Group, Centre for Haemato-Oncology, Barts Cancer Institute, Queen Mary University of London, London, UK; 3 Precision Medicine Group, Centre for Haemato-Oncology, Barts Cancer Institute, Queen Mary University of London, London, UK; 4 Flow Cytometry Core Facility, Barts Cancer Institute, Queen Mary University of London, London, UK; 5 Tissue Bank, Barts Cancer Institute, Queen Mary University of London, London, UK; 6 Department of Haematological Medicine, King’s College London School of Medicine, London, UK

Kinase inhibitors are efficient in reducing cancer cell viability in cases where malignant cells present a dependency or addiction to the targeted kinase [1]. Genetic alterations can cause constitutive activation of pro-survival and proliferative pathways and often determine the extent by which cancer cells respond to targeted drugs [2, 3]. However, other biochemical events, not directly linked to genetic mutations may also contribute to the modulation of oncogenic kinase activity and thus influence responses to kinase targeted drugs [4, 5]. Here, we integrated drug sensitivity, proteomic, phosphoproteomic, immunophenotypic, and genomic analyses of primary AML to rationalize responses and identify determinants of sensitivity of AML cells to targeted compounds of clinical and preclinical interest in this disease.

We investigated the effects on cell viability of inhibitors for the kinases FLT3/PKC (midostaurin), PAK (PF-3758309), CK2 (silmitasertib), MEK (trametinib), and P38 (TAK-715). Hereafter named as FLT3/PKCi, PAKi, CK2i, MEKi, and P38i, respectively. Dose–response curves for cells obtained from 36 AML patients (Data File S1) showed heterogeneous responses to all compounds (Figure S1). However, samples of the M4 FAB subtype were on average more sensitive than M1 samples to MEKi (Fig. 1a).

**Fig. 1:**
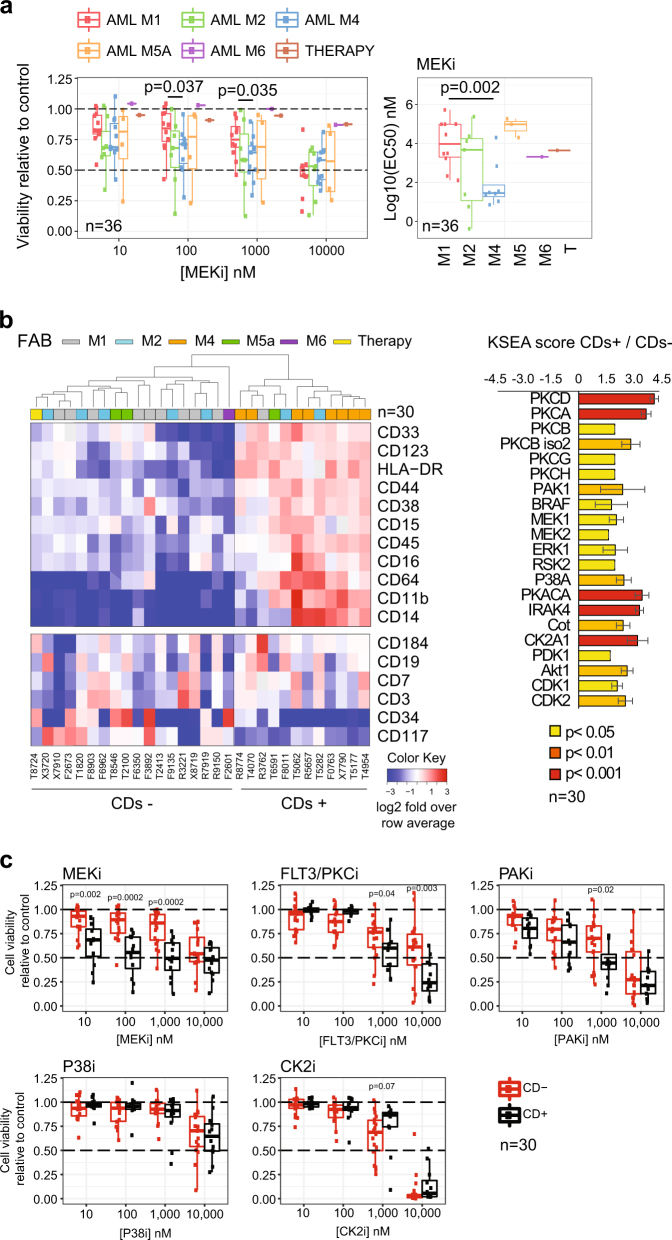
Association of differentiation, kinase activity, and sensitivity to kinase inhibitors in primary AML. **a** Sensitivity to MEKi as a function of FAB group. **b** CD expression across 30 cases and estimation of individual kinase activities in CDs+ and CDs− groups. **c** Sensitivity to kinase inhibitors as a function of CD pattern expression. Significance was assessed by Mann–Whitney test in **a**, **c** and with a *z*-test in **b**

Based on the surface expression of a set of co-expressed CD markers (Figure S2a), mass cytometry data subdivided our patient samples into two groups (Fig. 1b (heatmap)). These analyses could be performed in 30 cases with sufficient number of cells and produced two main groups, which we termed CDs+ and CDs−, consisting of 12 and 18 patients, respectively. Untargeted mass spectrometry proteomics (Data File S2) uncovered greater expression in the CDs+ group relative to CDs− of a set of proteins linked to differentiation, several kinases, and other signal-transduction regulators (Figure S2b, c). Global phosphoproteomics by mass spectrometry showed that CDs+ cells had an increase in protein phosphorylation relative to the CDs− cases (Figure S2d, Data File S3) and activated kinases downstream growth factor signaling, as illustrated by kinase substrate enrichment analysis [6] (Fig. 1b (bar plot)). In addition, individual phosphorylation markers [7] on ERK1/2 (MAPK3/1), PAK1/2, MEK1 (MAP2K1), and PKCδ (PRKCD) were highly phosphorylated in the CDs+ group (Figure S2d) and correlated with the surface expression of individual CD markers linked to differentiation (Figure S3a and S3b).

Since CDs+ cases activated kinase survival pathways to a greater extent than CDs− cases, we reasoned that cells from these groups would respond differently to kinase inhibitors. Consistently with this hypothesis, cell viability analysis as a function of treatment with kinase inhibitors showed that CDs+ cases were more sensitive than CDs− to MEKi (at 10, 100, and 1000 nM), FLT3/PKCi (1 and 10 μM), and PAKi (1 μM) (Fig. 1c). These concentrations are physiologically relevant for MEKi and FLT3/PKCi [8, 9]. Together, our results suggest that CDs+ cells had higher expression of proteins associated with myelomonocytic differentiation and kinase signaling relative to negative cells, and consequently showed high phosphorylation and activation of pro-survival kinases, which was translated into an increased sensitivity to treatments with PAKi, midostaurin, and trametinib.

In order to rationalize drug responses with greater detail, we sequenced 25 genes frequently mutated in AML in 27 cases of our cohort (Data File S4, sequencing failed in three samples). We found that genes involved in kinase signaling (*NRAS*, *BRAF*, and *FLT3*), were more frequently mutated in CDs+ cases (Figure S4, *p* = 0.008 by hypergeometric test). We performed an integrative and systematic analysis of mutational profiles with the mass spectrometry and cytometry data. Cells positive for *NRAS* mutations, high MAPK1 phosphorylation, or the CDs+ phenotype were more sensitive to MEKi than negative cells (Fig. 2a (i–iv)). Cells with the *NRAS/BRAF/FLT3-ITD* genotypes were not more sensitive to MEKi than cells with just either *NRAS* or *BRAF* mutations (Fig. 2a (v)). In contrast, cases positive for *NRAS*, *BRAF* mutations or the CDs+ phenotype (*NRAS*/*BRAF*/CDs+) were on average more sensitive to MEKi than cells without this molecular signature (Fig. 2a (vi–ix)). The *p* value assessment for the comparisons showed that the *NRAS*/*BRAF*/CDs+ signature produced the most significant difference followed by the NRAS/BRAF/p-MAPK1hi/CDs+ signature (Fig. 2 (bar plot)). Our results suggest that, in addition to *NRAS/BRAF* activating mutations, the RAS/MEK/ERK pathway may be activated by other means in cells with high expression of CD markers. Thus, MEKi treatment was more likely to reduce AML cell viability in cases positive for at least one of these markers (NRAS/BRAF mutations or specific CD pattern expression).

**Fig. 2:**
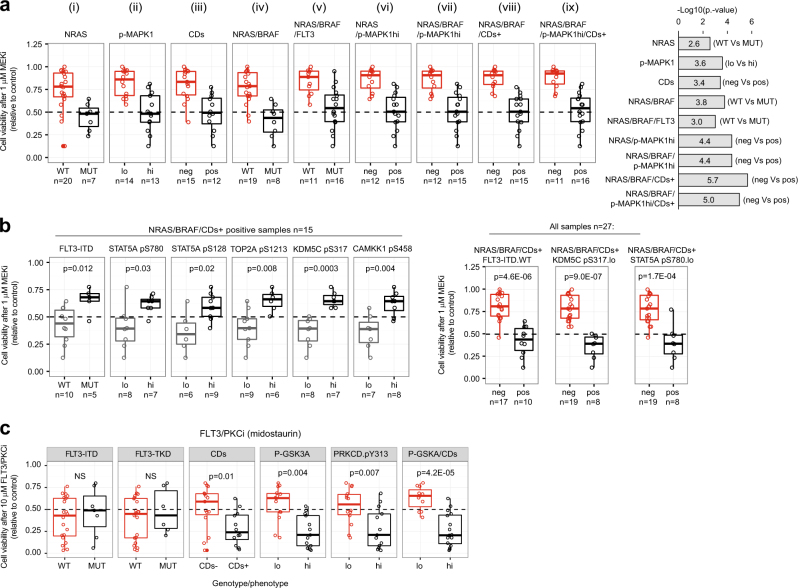
Integration of genomic, phosphoproteomics, and mass cytometry data to rationalize kinase inhibitors sensitivity. **a** Viability of AML cells within the indicated genotype/phenotype groups after treatment with MEKi. **b** Sensitivity of NRAS/BRAF/CDs+ positive cells to MEKi as a function of the indicated factors. **c** FLT3/PKCi sensitivity of AML cells with the indicated phenotype/genotype. Phosphorylations are denoted as (hi) and (lo) based on a greater or lower phosphorylation than the median across all cases. Significance was assessed by Mann–Whitney test

Although 15 cases with the *NRAS*/*BRAF*/CDs+ signature were on average more sensitive to MEKi than negative cases, 8 of such cases were resistant (viability >50%) to treatment (Fig. 2a (viii)). Within these 15 cases positive for *NRAS*/*BRAF*/CDs+, cells with *FLT3*-ITD mutations were significantly more resistant to MEKi than cells without this mutation (*p* = 0.012, Fig. 2b). Several phosphorylation markers were also found to be associated with responses to MEKi within the *NRAS*/*BRAF*/CDs+ cases, including STAT5A^S780^, STAT5A^S128^, TOP2A^S1213^, KDM5C^S317^, and CAMKK1^S458^ (Fig. 2b). When the whole cohort of 27 patients was considered, samples positive for *NRAS*/*BRAF*/CDs+ and negative for *FLT3*-ITD or low pSTAT5A or pKDM5C were more sensitive to MEKi than the other cells (Fig. 2d, Figure S5a). This higher sensitivity of *NRAS*/*BRAF*/CDs+ cases that were *FLT3*-ITD negative or pKDM5C^S317^ low was consistent across several MEKi concentrations (Figure S5a).

Our results suggest two distinct mechanisms of intrinsic resistance to MEK inhibition. One occurs in cells that are not addicted to the pro-survival actions of MEK because these have low RAS/MEK/ERK pathway activity. The other occurs in cells which, albeit having a highly active RAS/MEK/ERK, bypass MEK inhibition using the FLT3/STAT5 axis; a pathway known to sustain AML viability and proliferation by acting in parallel to RAS/MEK/ERK signaling [10, 11]. Pemovska et al. [12] also observed a high response to trametinib in a subgroup of AML primary cells. *NRAS* is frequently mutated in AML and in a recent clinical trial ~20% of AML patients positive for *NRAS* or *KRAS* mutations responded to trametinib [13]. Our results suggest that selection of patients for therapy based not only on *NRAS/KRAS* mutations but also on direct markers of MEK activity, and STAT5 and KDM5A phosphorylation may increase the proportion of patients that will respond to this treatment.

We also noted that *FLT3*-ITD status was not associated with the responses of cells to FLT3/PKCi (Fig. 2c, Figure S5b), an inhibitor recently approved to treat FLT3 mutant AML [14]. In contrast, CD expression and phosphorylation markers on PKCδ and on its substrate GSK3A [15] were increased in FLT3/PKCi-sensitive cells at 10 μM and 1 μM (Fig. 2c, Figure S5b). Our results suggest that the mode of action of midostaurin may involve the inhibition of PKCδ (a known target of this drug), which we found activated in primary AML (Fig. 1b, Figure S4a).

In conclusion, we found that AML cells remodel their kinase-signaling network during differentiation, resulting in a marked increase in the activity of pro-survival pathways regulated by MEK and PKC. Specific combinations of target and parallel kinase-pathway activation (caused by genetic and non-genetic events) determined the extent by which AML cells respond to treatments with trametinib or midostaurin.

## Electronic supplementary material

Supplementary information

Data Set 1

Data Set 2

Data Set 3

Data Set 4
