# Supplementary material for: Proteomic and genomic integration identifies kinase and differentiation determinants of kinase inhibitor sensitivity in leukemia cells
Source: Leukemia. 2018 Apr 7;32(8):1818–22. doi: 10.1038/s41375-018-0032-1 (PMC5949212; doi:10.1038/s41375-018-0032-1)
Supplement: Supplementary file 1 — Supplementary information [file 41375_2018_32_MOESM1_ESM.docx]

**Supplementary Information**

**Proteomic and genomic integration identifies kinase and differentiation determinants of kinase inhibitor sensitivity in leukemia cells**

Pedro Casado^1^, Edmund H. Wilkes^1^, Farideh Miraki-Moud^2^, Marym M. Hadi^1†^, Ana Rio-Machin^3^, Vinothini Rajeeve^1^, Rebecca Pike^4^, Sameena Iqbal^5^, Santiago Marfa^1††^, Nicholas Lea^6^, Steven Best^6^, John Gribben^2^, Jude Fitzgibbon^3^, Pedro R. Cutillas^1^*

Author Contributions

P.C. designed and conducted experiments, analysed and interpreted data, prepared figures, wrote the manuscript; E.H.W. performed experiments and edited manuscript; F.M-M. performed experiments; M.M.H performed experiments; A.R.M. performed DNA analyses; V.R. performed mass spectrometry experiments; R.P. performed Cy-TOF experiments; S.I. provided biobank management and analyzed patient data; S.M. performed experiments; N.L. performed DNA sequencing; S.B. performed DNA sequencing; G.M. performed DNA sequencing; J.G. provided critical analysis and edited manuscript; J.F. provided critical analysis; P.R.C. conceived study, designed experiments, performed bioinformatic experiments, analyzed and interpreted data, prepared figures, wrote manuscript.

Supplementary Data

**Contents**

Supplementary materials and methods

Supplementary Table 1. List of antibodies used for mass cytometry analysis

Supplementary Figure 1. Primary AML samples showed a heterogeneous response to kinase inhibitors.

Supplementary Figure 2. Differentiation markers selected based on correlation stratify patients in groups with different expression of proteins and phosphopeptides.

Supplementary Figure 3. Correlation between the phosphorylation of kinases at regulatory sites and the surface expression of the indicated differentiation markers

Supplementary Figure 4: Mutation in genes linked to kinase signaling are associated to differentiation stage and to specific phosphorylation patterns.

Supplementary Figure 5. Viability of AML cells with the indicated genotype/phenotype as a function of kinase inhibitor treatment

Supplementary References

Supplementary Data file 1. Individualized clinical and molecular features of AML patients

Supplementary Data file 2. Proteins identified and quantified in the AML primary cells

Supplementary Data file 3. Phosphopeptides identified and quantified in AML primary cells as a function of CDs groups

Supplementary Data file 4. Mutations identified in the AML primary cells and their associated phosphopeptides

Supplementary Materials and Methods

Study Design

The study was performed in 36 primary samples of mononuclear cells extracted from the peripheral blood of AML patients at diagnosis. Patients gave informed consent for the storage and use of their blood cells for research purposes. Experiments were performed in accordance with the Local Research Ethics Committee, as previous described ^1^. The clinical details of the 36 cases used in this study are shown in Supplementary Data file 1. In this pilot study power analysis was not performed to estimate sample sizes. Samples were randomly selected from the BCI tissue bank collection. Initially, 45 samples were included in the study but nine were later excluded because these were not viable in the *ex-vivo* experiments. Material availability allowed proteomics and mass cytometry analysis of 30 samples and DNA sequencing of 27 samples. Proteomics, phosphoproteomics and drug sensitivity analysis were performed before the stratification of the patients into CDs groups. Scientist performing the DNA analysis were blinded in respect to the allocation of the samples into the CDs groups.

*Viability assay*

Ex-vivo drug testing of AML primary cells was as previously described ^2^. Briefly, cells were re-suspended in MS-5 conditioned IMDM medium, seeded in 96 well plates and treated with vehicle or 1 to 10000 nM of the indicated inhibitors for 72h. Cells were stained with Guava ViaCount reagent and viability was measured. Flow cytometry data were analyzed using CytoSoft (v2.5.7). *Ex-vivo* drug testing was performed in quadruplicate sampling replicates and viability values averaged and expressed relative to vehicle control.

Mass Spectrometry

Cell vials were thawed at 37 C in a water bath for 10 min and cell suspensions were transferred to fresh tubes and incubated with DNAse for 5 min at 37 C. PBS supplemented with 2% FBS was added to the suspension, cells were centrifuged at 500xg at room temperature for 5 min and cell pellets were resuspended in IMDM medium supplemented with 10% FBS and 1% penicillin/streptomycin. After cell counting, 10 mL of cell suspension were seeded in T75 flask (15x10^6^ cells/mL) and maintained in an incubator for 2h at 37 C and 5% CO_2_. Cells were lysed and proteins digested using trypsin as previously described ^3^. Cell were harvested by centrifugation at 500xg at 4 ^o^C for 5 min, washed twice with cold PBS supplemented with 1mM Na_3_VO_4_ and 1 mM NaF, snap frozen and stored at -80C until further processing. Cell pellets were lysed in urea buffer (8M urea in 20 mM in HEPES pH 8.0 supplemented with 1 mM Na_3_VO_4_, 1 mM NaF, 1mM Na_4_P_2_O_7_ and 1 mM sodium β-glycerophosphate) for 30 min and further homogenized by sonication (60 cycles of 30s on 40s off; Diagenode Bioruptor® Plus, Liege, Belgium). Insoluble material was removed by centrifugation at 20.000 x g for 10 min at 5 ^o^C and protein in the cell extracts was quantified by bicinchoninic acid (BCA) analysis.

For phosphoproteome analyses, we used published methods ^4-6^ with some modifications. Briefly, 250 µg of protein were reduced and alkylated by sequential incubation with 10 mM DTT and 16.6 mM iodoacetamyde for 1h. The urea concentration was diluted to 2M with 20 mM HEPES (pH 8.0) and 80 µL of conditioned trypsin beads [(50% slurry of TLCK-trypsin (Thermo-Fisher Scientific; Cat. #20230)] conditioned with 3 washes of 20 mM HEPES (pH 8.0)) were added and the samples incubated for 16h at 37 ^o^C with agitation. Trypsin beads were removed by centrifugation at 2,000 x g for 5 min at 5 ^o^C. For phosphoproteomics analyses, 100 µg of protein were used.

Following trypsin digestion, peptide solutions were desalted using 10 mg OASIS-HLB cartridges (Waters, Manchester, UK). Briefly, OASIS cartridges were accommodated in a vacuum manifold (-5 mmHg), activated with 1 mL ACN and equilibrated with 1.5 mL washing solution (1% ACN, 0.1% TFA). After loading the samples, cartridges were washed with 1 mL of washing solution. For phosphoproteomics analyses, peptides were eluted with 500 µL of glycolic acid buffer 1 (1 M glycolic acid, 50% ACN, 5% TFA) and subjected to phosphoenrichment. For proteomics analyses peptides were eluted with 500 µL of ACN solution (30% ACN, 0.1% TFA), dried in a speed vac (RVC 2-25, Martin Christ Gefriertrocknungsanlagen GmbH, Osterode am Harz, Germany) and stored at -80 ^o^C.

Phosphopeptides were enriched using TiO_2_ (GL Sciences) as previously described with some modifications ^3^. Sample volumes were normalized to 1 mL using glycolic acid buffer 2 (1 M glycolic acid, 80% ACN, 5% TFA), 50 µL of TiO_2_ beads (50% slurry in 1% TFA) were added to the peptide mixture, incubated for 5 min at room temperature with agitation and centrifuged for 30s at 1500xg. For each sample, 80% of the supernatant was transfer to fresh tubes and stored in ice and the remaining 20% used to resuspend the bead pellets that were loaded into an empty prewashed PE-filtered spin-tips (Glygen, MD, USA) and packed by centrifugation at 1500 x g for 3 min. After loading the remaining volume of the supernatant by centrifugation at 1500xg for 3 mim, spin tips were sequentially washed with 100 µL of glycolic acid buffer 2, ammonium acetate buffer (100 mM ammonium acetate in 25% ACN) and 10% ACN by RT centrifugation for 3 min at 1500xg. For phosphopeptide recovery, the addition 50 µL of 5% ammonium water followed by centrifugation for 5 min at 1500 x g was repeated 4 times. Eluents were snap frozen in dry ice, dried in a speed vac and peptide pellets stored at -80 ^o^C.

For phosphoproteomics, peptide pellets were resuspended in 12 µL of reconstitution buffer (20 fmol/µL enolase in 3% ACN, 0.1% TFA) and 5.0 µL were loaded onto an LC-MS/MS system consisting of a Dionex UltiMate 3000 RSLC directly coupled to an Orbitrap Q-Exactive Plus mass spectrometer (Thermo Fisher Scientific). For proteomics, pellets were resuspended in reconstitution buffer (0.5 µg/µL) and 2 µL were injected. The LC system used mobile phases A (3% ACN: 0.1% FA) and B (100% ACN; 0.1% FA). Peptides were trap in a μ-pre-column (catalog no 160454) and separated in an analytical column (Acclaim PepMap 100 ;catalog no 164569). The following parameters were used: 3% to 23% B gradient for 120 min and a flow rate of 0.3 µL/min. Samples were run in the LC-MS/MS system in a randomized manner by shuffling samples before loading.

As they eluted from the nano-LC system, peptides were infused into the online connected Q-Exactive Plus system operating with a 2.1s duty cycle. Acquisition of full scan survey spectra (m/z 375-1,500) with a 70,000 FWHM resolution was followed by, data-dependent acquisition in which the 20 most intense ions were selected for HCD (higher energy collisional dissociation) and MS/MS scanning (200-2,000 m/z) with a resolution of 17,500 FWHM. A 30 s dynamic exclusion period was enabled with an exclusion list with 10 ppm mass window. Overall duty cycle generated chromatographic peaks of approximately 30 s at the base, which allowed the construction of extracted ion chromatograms (XICs) with at least 10 data points. The mass spectrometry proteomics data have been deposited to the ProteomeXchange Consortium via the PRIDE ^7^ partner repository with the dataset identifier PXD005978 and DOI 10.6019/PXD005978.

Mascot Daemon 2.5.0 was used to automate peptide identification from MS data. Peak list files (MGFs) from RAW data were generated with Mascot Distiller v2.5.1.0 and loaded into the Mascot search engine (v2.5) in order to match MS/MS data to peptides **^8^**. The searches were performed against the SwissProt Database (SwissProt_2012Oct.fasta for proteomics or uniprot_sprot_2014_08.fasta for phosphoproteomics analysis) with a FDR of ~1% and the following parameters: 2 trypsin missed cleavages, mass tolerance of ±10 ppm for the MS scans and ±25 mmu for the MS/MS scans, carbamidomethyl Cys as a fixed modification, PyroGlu on N-terminal Gln and oxidation of Met as variable modifications. For phosphoproteomics experiments Phosphorylation on Ser, Thr, and Tyr was also included as variable modifications. The in-house developed Pescal software was used for label-free peptide quantification **^9^**, XICs for all the peptides identified across all samples were constructed with ±7 ppm and ±2 min mass and retention time windows, respectively. Peak areas from all XICs were calculated. Undetectable peptides were given an intensity value of 0. Values of 2 technical replicates per sample were averaged and intensity values for each peptide were normalized to total sample intensity.

Mass Cytometry

Mass cytometry was used to characterize CD markers in AML cells ^10^. Primary cells (4x10^6^) were transferred to fresh tubes, washed twice with PBS and incubated with 1x Cell-ID™ Cisplatin solution (Fluidigm; Cat. 201064) for 5 min at RT. Cells were washed with Maxpar Cell Staining buffer and pellets were resuspended and incubated with 50 µL of 20 μg/mL HAG (human γ-Globulins, Sigma-Aldrich; Cat. G4386-1G) for 20 min at RT. After adding 50 µL of antibody mix (1/50 dilution of each antibody; Supplementary Table 1), samples were incubated for 30 min at RT. The cells were then washed twice with Maxpar Cell Staining buffer, pellets were resuspended in Fix and Perm Buffer and left overnight at 4 ^o^C. Next day, Ir intercalator was added to a final concentration of 1x and samples were incubated for 20 min at RT. Permeabilized cells were washed twice with Maxpar Cell Staining buffer and twice with Maxpar water. Samples were analyzed on a CyTOF2 mass cytometer (Fluidigm). Data were normalized using the normalizer within the DVS Sciences CyTOF Instrument Control Software (v 6.0.626).

Panel Sequencing

Target enrichment of a 25 gene myeloid panel was achieved using an in-house True SeqCustom Amplicon (TSCA) design (Illumina, San Diego, USA). Genes included in this panel are shown in Supplementary Data 7.

Sanger Sequencing

Primers for BRAF V600 PCR were forward 5’-TCTTCATGAAGACCTCACAGT-3’ and reverse 5’-CCAGACAACTGTTCAAACTGA-3’. 20-50 ng of DNA was used as template and the thermal conditions were as follows: initial heating period for 15 min at 95°C, 36 cycles at 95°C for 1 min, 55°C for 1 min and 72°C for 1 min, and finally 10 min at 72°C. Amplicons were sequenced by GATC Biotech (Constanza, Germany) using the forward primer. Positive cases were validated using the reverse primer.

Bioinformatics

Inference of kinase activities from the phosphoproteomics data was performed using Kinase substrate enrichment analysis (KSEA) as described before ^2^. DAVID software (https://david.ncifcrf.gov/) was used to determine the enrichment of gene ontologies (GO), which were considered enriched when the Bonferroni’s corrected p-values were < 0.05. Hierarchical clusters were constructed within the R statistical computing environment (3.2.3) using the Euclidean distance metric in the heatmap2 package. EC50 were calculated in R from the cell viability data using the DRC package (W1.4 function). Statistical analysis was performed in R (version 3.2.3) or Microsoft Excel 2013. Unpaired, two-tail Student’s t-test was used to assess significance in phosphoproteomics data. Normalized intensity values were assumed to follow a normal distribution, groups were assumed to present similar variance and p-values were adjusted for multiple testing using Benjamini-Hochberg procedures. Two-tail Mann-Witney test was used to assess significance in cell viability data, while hypergeometric test was used to calculate enrichment in KSEA analysis.

Code Availability

For access to the code of KSEA algorithm contact the corresponding author.

Authorship

P.C. designed and conducted experiments, analysed and interpreted data, prepared figures, wrote the manuscript; E.H.W. performed experiments and edited manuscript; F.M-M. performed experiments; M.M.H performed experiments; A.R.M. performed DNA analyses; V.R. performed mass spectrometry experiments; R.P. performed Cy-TOF experiments; S.I. provided biobank management and analysed patient data; S.M. performed experiments; N.L. performed DNA sequencing; S.B. performed DNA sequencing; G.M. performed DNA sequencing; J.G. provided critical analysis and edited manuscript; J.F. provided critical analysis; P.R.C. conceived study, designed experiments, performed bioinformatic experiments, analysed and interpreted data, prepared figures, wrote manuscript.

**Supplementary Table 1. List of antibodies used for mass cytometry analysis.** All antibodies were obtained from FLUIDIGM.

| **Antigen** | **Determinant** | **Clone** | **Metal** | **Catalogue** |
| --- | --- | --- | --- | --- |
| CD19 | Co-receptor for CD21 | HIB19 | 142Nd | 201316 |
| CD117 | Receptor Tyrosine Kinase /SCF | 104D2 | 143Nd | 201316 |
| CD11b | Fibrinogen Receptor | ICRF44 | 144Nd | 201316 |
| CD64 | Fc Receptor | 10.1 | 146Nd | 201316 |
| CD7 |  | CD7-6B7 | 147Sm | 201316 |
| CD123 | Interleukin 3 Receptor | 6H6 | 151Eu | 201316 |
| CD45 | Receptor Tyrosine Phosphatase | HI30 | 154Sm | 201316 |
| CD33 | Sialic Acid Receptor | WM53 | 158Gd | 201316 |
| CD15 | Carbohydrate | W6D3 | 164Dy | 201316 |
| CD34 | Cell-Cell Adhesion Factor | 581 | 166Er | 201316 |
| CD3 | TCR Co-receptor | UCHT1 | 170Er | 201316 |
| CD44 | Hyaluronic Acid Receptor | IM7 | 171Yb | 201316 |
| CD38 | Synthesis of Cyclic ADP | HIT2 | 172Yb | 201316 |
| HLA-DR | Antigen Presentation | L243 | 174Yb | 201316 |
| CD184 | Chemokine Receptor /PSD-1 | 12G5 | 175Lu | 201316 |
| CD14 | Co-activator of TLR4 | M5E2 | 160Gd | 3160001B |
| CD16 | Fc Receptor | 3G8 | 148Nd | 3148004B |

**Supplementary Figure 1. Primary AML samples showed a heterogeneous response to kinase inhibitors.** **(a)** Dose response curves for cell viability of each primary AML sample treated with a panel of kinase inhibitors. Data points are mean ± SD (n=3). **(b)** Clustering analysis of AML primary cells based on their EC50 to the named compounds. Patient biopsies (n=36) were obtained by the Barts Cancer Institute biobank with ethical consent.

**Supplementary Figure 2. Differentiation markers selected based on correlation stratify patients in groups with different expression of proteins and phosphopeptides.** (a) Pearson coefficients showing the correlated expression of multiple differentiation marker in our cohort of patients (n=30). **(b)** Overview of proteins overexpressed across CDs groups **(c)** Heatmap showing proteins whose expression is significantly increased (p-value < 0.05) in any of the CDs groups. **(d)** Overview of phosphopeptide abundances across the CDs groups.. **(e)** Heatmap showing phosphopeptides comprised in kinases whose expression is significantly increased in any of the CDs groups. In **(b)** and **(d)** p-values were calculated using unpaired, two-tailed Student’s t-test and adjusted following the Tukey or Benjamini-Hochberg procedures

**Supplementary Figure 3. Correlation between the phosphorylation of kinases at regulatory sites and the surface expression of the indicated differentiation markers. (a)** Correlation between the phosphorylation of MAPK1^Y187^ PAK2^S141^ and PRKCD^Y313^ and the surface expression of the indicated differentiation markers. **(b)** Significance of association between phosphomarkers indicated in section a and CD marker surface expression across AML cases (n=30).

**Supplementary Figure 4: Mutation in genes linked to kinase signaling are associated to differentiation stage and to specific phosphorylation patterns.** Mutations for the indicated genes across AML samples. Kinase sig. stands for any activating mutations in either FLT3, NRAS or BRAF. Risk was assigned based on ELM classification described by Döhner et al.^11^ Karyotype details are as follows: K1=46,XX,t(8;21)(q22;q22); K2=46,XY,t(15;16)(q26;q12.2~22)[50].ish_der(15)(CBFB+); K3=+8; K4=+3+10; K5=add(21p11.2); K6=47,XX,+13[6]/46,XX[4]; K7=t(6;11)(q27;q23); K8=-7; K9=Complex Inc. t(2;3)(q16:q26); K10=del(7q),+8,+(11q); K11=46,XX,dic(7;22)(q11.2;q10),+8.

**Supplementary Figure 5.** **Viability of AML cells with the indicated genotype/phenotype as a function of kinase inhibitor treatment. (a)** Viability of AML primary cells after treatment with MEKi. **(b)** Mann Whitney p-values for the viability after treatment with the indicated inhibitor in the indicated genotype/phenotype. F/Pi indicates FLT3/PKCi and MAPKs indicates MAPK1 or MAPK3. **(c)** Viability of AML cells after treatment with FLT3/PKCi. Two tailed Mann Whitney test was used to assess p-values; * p < 0.05; ** p < 0.01; *** p < 0.001, **** p < 0.0005.

**Supplementary References**

1. Stevens J, Waters R, Sieniawska C, Kassam S, Montoto S, Fitzgibbon J*, et al.* Serum selenium concentration at diagnosis and outcome in patients with haematological malignancies. *Br J Haematol* 2011 Aug; **154**(4)**:** 448-456.

2. Casado P, Rodriguez-Prados JC, Cosulich SC, Guichard S, Vanhaesebroeck B, Joel S*, et al.* Kinase-substrate enrichment analysis provides insights into the heterogeneity of signaling pathway activation in leukemia cells. *Sci Signal* 2013 Mar 26; **6**(268)**:** rs6.

3. Wilkes EH, Terfve C, Gribben JG, Saez-Rodriguez J, Cutillas PR. Empirical inference of circuitry and plasticity in a kinase signaling network. *Proc Natl Acad Sci U S A* 2015 Jun 23; **112**(25)**:** 7719-7724.

4. Gruhler A, Olsen JV, Mohammed S, Mortensen P, Faergeman NJ, Mann M*, et al.* Quantitative phosphoproteomics applied to the yeast pheromone signaling pathway. *Molecular & cellular proteomics : MCP* 2005 Mar; **4**(3)**:** 310-327.

5. Larsen MR, Thingholm TE, Jensen ON, Roepstorff P, Jorgensen TJ. Highly selective enrichment of phosphorylated peptides from peptide mixtures using titanium dioxide microcolumns. *Molecular & cellular proteomics : MCP* 2005 Jul; **4**(7)**:** 873-886.

6. Montoya A, Beltran L, Casado P, Rodriguez-Prados JC, Cutillas PR. Characterization of a TiO(2) enrichment method for label-free quantitative phosphoproteomics. *Methods* 2011 Aug; **54**(4)**:** 370-378.

7. Vizcaino JA, Csordas A, Del-Toro N, Dianes JA, Griss J, Lavidas I*, et al.* 2016 update of the PRIDE database and its related tools. *Nucleic Acids Res* 2016 Dec 15; **44**(22)**:** 11033.

8. Perkins DN, Pappin DJ, Creasy DM, Cottrell JS. Probability-based protein identification by searching sequence databases using mass spectrometry data. *Electrophoresis* 1999 Dec; **20**(18)**:** 3551-3567.

9. Cutillas PR. Targeted In-Depth Quantification of Signaling Using Label-Free Mass Spectrometry. *Methods Enzymol* 2017; **585:** 245-268.

10. Bandura DR, Baranov VI, Ornatsky OI, Antonov A, Kinach R, Lou X*, et al.* Mass cytometry: technique for real time single cell multitarget immunoassay based on inductively coupled plasma time-of-flight mass spectrometry. *Analytical chemistry* 2009 Aug 15; **81**(16)**:** 6813-6822.

11. Dohner H, Estey E, Grimwade D, Amadori S, Appelbaum FR, Buchner T*, et al.* Diagnosis and management of AML in adults: 2017 ELN recommendations from an international expert panel. *Blood* 2017 Jan 26; **129**(4)**:** 424-447.
